# Laboratory-selected, probiotic *Ligilactobacillus salivarius* SBT2687 supplementation is associated with subjective knee symptoms and the gut microbiome in adults with pre-osteoarthritis: a randomized controlled trial in Japanese

**DOI:** 10.3389/fnut.2026.1863405

**Published:** 2026-08-04

**Authors:** Erina Ushizawa, Takehiko Yasueda, Ayatake Nakano, Tomohiro Sugino, Masahiro Fukuda, Hiroshi M. Ueno

**Affiliations:** 1 Milk Science Research Institute, Megmilk Snow Brand Co., Ltd., Kawagoe, Japan; 2 Soiken Inc., Toyonaka, Japan; 3 Fukuda Clinic, Osaka, Japan

**Keywords:** gut microbiome, knee osteoarthritis, *Ligilactobacillus salivarius*, pre-osteoarthritis, probiotics, randomized controlled trial

## Abstract

**Introduction:**

Although the gut microbiome is involved in the pathogenesis of knee osteoarthritis (OA), the effects of dietary supplementation remain controversial. Probiotics are a promising option for lifestyle management of inflammatory diseases. This study aimed to investigate the effects of dietary supplementation with probiotics on subjective symptoms and the gut microbiome in adults with knee pre-OA.

**Methods:**

For the *in vitro* laboratory selection of probiotic strains, 110 strains of lactic acid bacteria and bifidobacteria were serially examined for expression profiles of OA-related genes in interleukin-1β–stimulated SW982 cells. The clinical trial was a double-blind, placebo-controlled, randomized clinical trial of Japanese adults with knee pre-OA based on Kellgren–Lawrence grade 0 or 1 and subjective symptoms. Probiotics or a placebo were administered during a 12-week intervention from January to April 2024. The primary outcomes were pain, stiffness, and discomfort in both knees, assessed using the visual analog scale (VAS). Secondary outcomes included subjective assessments, gut microbiome, blood biochemistry, and safety profiles, including adverse events.

**Results:**

*Ligilactobacillus salivarius* SBT2687 (LS2687) was identified in the analysis of gene expression profiles of SW982 cells. For the clinical trial, 108 participants (median age 52.0 years [IQR 47.0–57.0], 65.7% female) were included in the study. A greater reduction was observed in VAS scores for all symptoms in the LS2687 group than in the placebo group at 12 weeks (pain: −2.9 ± 6.1 vs. −1.7 ± 4.8, *P* = 0.048; stiffness: −3.6 ± 7.1 vs. −1.3 ± 5.1, *P* = 0.014; discomfort: −3.8 ± 7.5 vs. −1.7 ± 5.3, *P* = 0.016), although the between-group improvements did not survive baseline adjustment (ANCOVA *P* = 0.14–0.24). The species-level relative abundance of *Lactobacillus salivarius* increased in the LS2687 group (from 3.63% to 35.49%; *P* < 0.001 and 0.005 in within- and between-group comparisons, respectively). No adverse events were associated with this intervention throughout the study.

**Discussion:**

LS2687 supplementation was well-tolerated and provided new insights into the probiotic management of knee pre-OA. The gut-knee axis should be considered in the management of symptoms with knee pre-OA.

**Clinical Trial Registration:**

https://center6.umin.ac.jp/cgi-open-bin/ctr/ctr_view.cgi?recptno=R000059864, identifier [R000059864].

## 1 Introduction

Pre-osteoarthritis (pre-OA) refers to the early stages of joint degeneration, biochemical changes, and early structural abnormalities detected using advanced imaging techniques, with no overt clinically established OA symptoms (1). Pre-OA is characterized by the presence of risk factors, potential biomarkers, and early signs of clinical symptoms (2), as well as recently described as early-stage symptomatic knee OA [EsSKOA, (3)]. This stage represents a critical window for intervention, as treatments applied during this period may potentially prevent or significantly delay the progression to OA (4). Dietary management and supplementation provide building blocks, anti-inflammatory nutrients, antioxidants, and other food components that support the cartilage or directly support structural integrity of the joint (5). Emerging evidence on the use of dietary supplements is promising; however, establishing their clinical relevance is challenging, and study outcomes remain inconsistent (6, 7).

Both preclinical and clinical studies suggest that probiotics have demonstrated promising effects on pain relief and joint function in OA by modulating the gut microbiome and alleviating local and systemic low-grade chronic inflammation (8, 9). The concept of the “gut-joint axis” hypothesis represents a paradigm shift in our understanding of OA pathogenesis (10–12). This framework recognizes the bidirectional relationship between the gut microbiome – the complex community of microorganisms in the gastrointestinal tract – and joint health (13). Probiotic supplementation modifies the gut-joint axis by influencing gut microbiome composition, producing anti-inflammatory mediators, including short-chain fatty acids, as well as decreasing the production of pro-inflammatory cytokines, such as tumor-necrosis-factor (TNF)-α and interleukin (IL)-1β (14, 15), and interactions with immune cells in the gut. Nevertheless, findings from clinical studies are limited in terms of the exploratory roles of probiotics in OA, whereas preclinical studies have demonstrated the efficacy of probiotics in OA treatment. Different strains within the same species exert distinct clinical effects, and probiotics are characterized by strain and disease-specificity (16, 17). Although some probiotic strains have been identified as promising for OA management (18, 19), the literature is still limited, and the supporting evidence remains inconclusive. Therefore, further research is needed to identify and characterize probiotic strains for OA management. To study an earlier stage of OA in the context of disease prevention, we investigated effects of probiotics on individuals experiencing OA-related knee symptoms at an early structural stage without presence of established structural changes (3).

In this study, we aimed to investigate lactic acid bacteria with promising effects on improving pre-OA–related symptoms.

## 2 Materials and methods

### 2.1 *In vitro* identification of a probiotic bacterial strain using a human synovial cell line

We performed two-stage screening in lactic acid bacteria and bifidobacteria from 46 species 110 strains (Supplementary Tables 1 and 2) to screen the candidate species and strains in the first and second stages. Briefly, the human synovial cell line SW982 (American Type Culture Collection, Manassas, VA, USA) was cultured in Leibovitz-15 (L-15) medium (Sigma-Aldrich, St. Louis, MO) with 10% fetal bovine serum (FBS) and 1% a penicillin-streptomycin (Thermo Fisher Scientific, Waltham, MA, USA) at 37 °C in a humidified atmosphere containing 5% CO_2_. Cells (1.0 × 10^5^ cells/well in 12-well plates) were cultured for 7 days, switched to fresh serum-free L-15, and stimulated with human interleukin (IL-1β (1 ng/mL) for 24 h. Then, cell lysates of the lactic acid bacteria and bifidobacteria in the first stage, including *Lactobacillus salivarius (Lb. salivarius)* and *Lactococcus lactis (Lc. Lactis)* in the second stage – obtained from Megmilk Snow Brand (Tokyo, Japan), prepared from lyophilized culture using bead pulverization (5 × 60 s at 2,500 rpm with 60 s intervals), and resuspended in phosphate-buffered saline (PBS) to 20 mg/mL – were added at a final concentration of 1 mg/mL. Controls comprised blank (no IL-1β), negative control (IL-1β alone), and positive control (IL-1β plus 100 nM dexamethasone) in triplicates. Total RNA was extracted, reverse-transcribed, and quantified by THUNDERBIRD SYBR qPCR Mix and ViiA7 Real-time PCR system, using glyceraldehyde-3-phosphate dehydrogenase as the endogenous control. The cycling conditions were 95°C for 1 min; 95 °C for 15 s and 60 °C for 30 s; followed by melt-curve analysis with a 0.05°C/s ramp. Relative gene expression was calculated from standard curves. The sequences of all the primers used in this study are listed in Supplementary Table 3; the detailed experimental procedures are available in the Supplementary methods.

### 2.2 Clinical study

#### 2.2.1 Ethics

This study was performed in accordance with the provisions of the Declaration of Helsinki (revised in 2013) and the Ethical Guidelines for Life Sciences and Medical Research Involving Human Subjects stipulated by the Japanese government. The local research ethics committee approved the study protocol (Ref. YMC-2023-09). The study was prospectively registered at the University Hospital Medical Information Network on December 31, 2023 (https://center6.umin.ac.jp/cgi-open-bin/ctr/ctr_view.cgi?recptno=R000059864). All participants provided written informed consent.

#### 2.2.2 Study design and participants

This was a double-blind, placebo-controlled, randomized controlled trial conducted in Osaka, Japan. Two groups receiving *Ligilactobacillus salivarius* SBT2687 (LS2687) and placebo capsules were compared in parallel with a dietary intervention of three capsules administered daily at any time for 12 weeks. Outcome measures were examined from baseline to the end of the intervention, and the frequency was dependent on the nature of each measurement, as described in the Outcome Measures section.

Participants were recruited from the registered participant database of the clinical trial. The inclusion criteria were adults aged 30–65 years with knee pain and Kellgren and Lawrence (KL) grades 0–1 at recruitment and baseline measurement. The most symptomatic knee was selected as the primary symptomatic knee for all subsequent assessments. Exclusion criteria included a medical history of clinically symptomatic OA, rheumatoid arthritis, joint surgery of the knees, and any other medical history in both knees, including any medical treatment and the use of dietary supplements for knee pain relief at recruitment and baseline measurements. Regarding dietary criteria, individuals with dietary habits of frequent (≥3 days per week) consumption of foods containing high lactic acid bacteria and bifidobacteria, such as health foods and pharmaceuticals, were excluded. In addition, participants were excluded if they were deemed inappropriate for this study by the study investigators for any reason, had participated or planned to participate in another clinical study within 3 months of recruitment in this study, were potentially allergic to milk and dairy foods, or had a recent history of a large amount of blood collection and donation, raising safety concerns. The participants did not report any pain at baseline were also excluded from data analysis due to zero signal in the outcome measurement.

#### 2.2.3 Randomization and blinding

Eligible participants were randomized into the LS2687 and placebo groups, using a computer-generated sequence. A computer program randomly issued an identifier for the participants, and the participants were allocated in turns according to the identifiers. The allocation sequence was generated and held by Statcom Co., Ltd., which was not involved in conducting the study. The allocation sequence was concealed until completed primary analysis and was not accessible to investigators or participants throughout the study period. Data curation and interpretation were performed after unblinding, based on a prespecified analysis plan. Participants were enrolled by the investigators. Blinding and allocation procedures were conducted by Statcom. The allocation manager confirmed that the groups were not statistically different in terms of sex, age, KL grades, and intensities of knee pain, stiffness, and discomfort at rest assessed using visual analog scale (VAS); and the knee pain scale assessed using the Japanese Knee Osteoarthritis Measure (JKOM) questionnaire.

The participants were informed that they would be randomized to one of two groups: outcome assessors, clinicians, or other healthcare professionals who performed the study were blinded to the allocation.

#### 2.2.4 Test foods

Two types of capsules, LS2687 and a placebo, were prepared as test foods. The test foods were manufactured by API Co., Ltd. (Gifu, Japan). In the LS2687 group, the capsules contained more than 1 × 10^11^ cells/g of LS2687, whereas the placebo group contained tapioca starch. The test foods were indistinguishable in terms of color, shape, and taste. The nutritional composition is summarized in Supplementary Table 4.

#### 2.2.5 Primary outcomes

The primary outcome was change from baseline to week 12 in knee pain-related intensity, defined as “the average levels of pain, stiffness, and discomfort in your knee today” measured on a 100-mm visual analog scale (VAS), with 100 being the worst imaginable intensity of pain, stiffness, and discomfort. Pain-related intensity was registered daily, and the participants were further asked to record the intensity upstairs, downstairs, locomotion, wakeup, and bedtime at rest and sitting in a kneeling position in a paper diary throughout the intervention period.

#### 2.2.6 Secondary outcomes

Secondary outcomes included subjective assessments, blood biochemistry analyses, and gut microbiome analyses. We used the JKOM and Japanese Orthopaedic Association Score for Osteoarthritic Knees (JOA) for subjective evaluation. JKOM is a patient-based self-assessment that includes four related items that reflect the Japanese cultural lifestyle (20). JKOM questionnaire assesses subjective knee symptoms through participants’ answers on a VAS about the degree of knee pain. The four items consist of eight questions about “II: pain and stiffness conditions”, 10 questions about “III: condition in daily life”, five questions about “IV: general activities”, and two questions about “V: health conditions” for a total of 25 questions. Participants answered each question on a 5-point Likert scale ranging from 0 (no disability) to 4 (severe disability) for chronic symptoms in the past few days and health status in the past month. Items II–V were summed to give a total score of 0–100. JOA is a reliable and valid observer-based knee scoring tool for evaluating functional status in patients with knee OA (21). It consists of four domains with a maximum of 100 points: pain on walking (30 points), pain on ascending or descending stairs (25 points), mobility (35 points), and joint effusion (10 points).

#### 2.2.7 Gut microbiome analysis

Gut microbiome analysis was performed by a third-party laboratory (TechnoSuruga Laboratory Co., Ltd., Shizuoka, Japan). Fecal samples were collected using a commercial stool collection tube kit, and genomic DNA was extracted using the ISOSPIN Fecal DNA kit (Nippon Gene, Japan) with bead-beating (FastPrep-24 5G, MP Biomedicals, USA). For 16S rRNA gene amplicon sequencing, the V3–V4 region of the bacterial 16S rRNA gene was amplified and barcoded, and paired-end sequencing was performed on an Illumina MiSeq platform using the MiSeq Reagent Kit v3 (600-cycle). Primer sequences were removed using Cutadapt (v1.18) (22), and paired-end reads were merged using fastq-join (v1.3.1). Sequence data were processed in QIIME 2 (v2020.6) (23). Denoising was performed using DADA2 (v1.10.0) (24) to generate amplicon sequence variants (ASVs) and a feature table. Taxonomy was assigned in QIIME 2 using a Naive Bayes classifier trained on the SILVA database (v138) (25). Relative abundance at each taxonomic level (e.g., genus and species) was calculated from the feature table aggregated by taxonomic level. Alpha diversity was evaluated using the Shannon–Wiener index. The relative abundance of *Lb. salivarius* within the *Lactobacillus* genus was calculated by normalizing the relative abundance of *Lb. salivarius* to the total relative abundance of the *Lactobacillus* genus in each sample.

#### 2.2.8 Safety analysis

For safety assessments, medical interviews, physical measurements (height and weight), and physiological tests (blood pressure and pulse rate) were performed at screening and 0, 4, 8, and 12 weeks after the start of intake. In addition, hematological parameters (hemoglobin, hematocrit, white and red blood cell counts, blood platelets, mean corpuscular volume, mean corpuscular hemoglobin, and mean corpuscular hemoglobin concentration), blood biochemical parameters (total protein, albumin, albumin/globulin ratio, alkaline phosphatase, aspartate aminotransferase, alanine transaminase, lactate dehydrogenase, gamma-glutamyl transpeptidase, total bilirubin, total cholesterol, low-density lipoprotein cholesterol, high-density lipoprotein cholesterol, triglycerides, sodium, potassium, chloride, calcium, magnesium, phosphorus, blood urea nitrogen, creatinine, uric acid, glucose, hemoglobin A1c, and creatine phosphokinase), and urinalysis parameters (urine specific gravity, pH, ketones, occult blood, urobilinogen [qualitative], bilirubin [qualitative], protein [qualitative], and glucose [qualitative]) were measured at the same time. All measurements were performed by BML, Inc. (Tokyo, Japan). In addition, the participants were instructed to keep daily records of their intake of test samples, changes in lifestyle habits (including diet, alcohol consumption, and number of cigarettes smoked), menstrual cycles (for female participants), and medication use. Adherence to the intervention was monitored using daily records.

### 2.3 Statistical analysis

For the *in vitro* study, data are presented as means ± standard deviation (SD). Data were statistically analyzed using EZR (26). Differences between the negative control group and the other groups were assessed using one-way analysis of variance followed by Dunnett’s test. *P-*values < 0.05 were considered statistically significant.

In the clinical study, the target effect and variance were based on an estimate of the invasiveness, and burden associated with the intervention was taken into consideration. A sample size of 100 (approximately 50 participants per group) was calculated to enable us to detect a target difference of ≥6 mm in VAS pain at the two-sided 0.05 significance level (one comparison) and a SD of 10 mm corresponding to a moderate effect size (27).

All statistical analyses, including sensitivity and subgroup analyses, followed a prespecified analysis plan. Interim analyses were not performed. The analysis population included all randomized participants. The primary outcome, change in knee pain, stiffness, and discomfort on the VAS from baseline to week 12, was analyzed using the Mann–Whitney *U*-test. The secondary outcome measures, JKOM, JOA and gut microbiome, were compared between groups using the Mann–Whitney *U*-test according to the distribution. For dichotomous measures, Fisher’s exact test was applied for analyses between groups. Sensitivity analyses were performed for the primary outcome, specifically the change in knee pain on the VAS, with subgroup analyses performed to explore potential confounders, such as sex, age, body mass index (BMI), KL grade, and the relative abundance of *Lb. salivarius* in the gut microbiome. BMI thresholds were set at 25.0 kg/m^2^ and 23.0 kg/m^2^. The threshold of 25.0 kg/m^2^ was set according to the Japanese definition of obesity (28). The threshold of 23.0 was set to achieve an equal sample size. The KL Grade is a five-level grading system for assessing knee OA progression based on radiographs. Subgroup analysis was performed using the KL Grade of the dominant knee. For the gut microbiome, we included additional parameters on the composition of the gut microbiome in relation to the supplementation effect of LS2687, based on the hypothesis that LS2687 interacts with the gut microbiome and exerts treatment effects on the primary outcome. Crude regression models were also examined for sensitivity analyses on the baseline VAS scores and their changes during intervention. Analysis of covariance (ANCOVA) was performed as a confirmatory analysis for the VAS outcomes after adjusting with baseline VAS values based on the entire statistical results. All statistical analyses were performed using SPSS version 29. Participants with missing or non-calculable data were excluded from the respective analyses.

## 3 Results

### 3.1 Identification of the probiotic effects of lactic acid bacteria on gene expression in SW982 cells

For the screening experiment, SW982 cells were used to evaluate the expression of genes associated with OA progression and pain (*TNF-α, MMP-13, COX-2*, and *NGF*). In total, 110 strains of lactic acid bacteria and bifidobacteria (Supplementary Table 1) were prepared and evaluated during primary screening. Among them, three strains of *Lb. salivarius* and the three strains of *Lc. lactis* reduced the expression of joint-related genes, indicating a species-dependent effect (Supplementary Figure 1).

**FIGURE 1:**
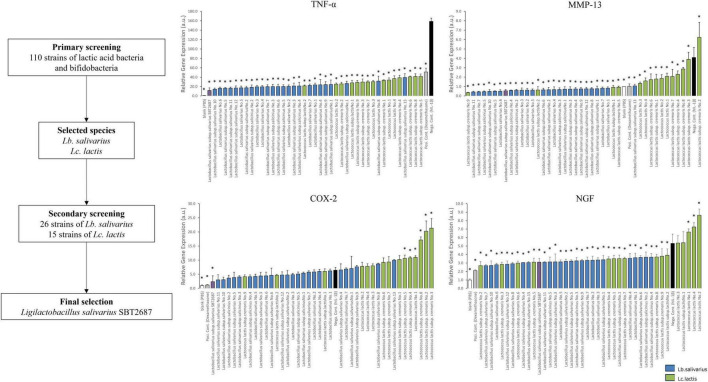
*In vitro* screening workflow and secondary screening results for the expression of joint-related genes. The left panel shows the screening workflow, from primary screening of 110 strains to final selection of *Ligilactobacillus salivarius* SBT2687. In the secondary screening, 26 strains of *Lactobacillus salivarius* and 15 strains of *Lactococcus lactis* were evaluated for their effects on the expression of joint-related genes. The SW982 cells were treated with interleukin (IL)-1β (1 ng/mL, negative control), dexamethasone (100 nM), and lactic acid bacteria samples (1 mg/mL). The expression levels of tumor necrosis factor (TNF)-α, matrix metalloproteinase (MMP)-13, Cycloxygenase (COX)-2, nerve growth factor (NGF) were measured. Data are shown as mean ± standard deviation (SD; *n* = 3) and were analyzed by one-way analysis of variance followed by Dunnett’s test (*P < 0.05 vs. negative control).

Based on these results, we hypothesized that these species would exhibit strong activity in reducing the expression of joint-related genes. Therefore, 26 strains of *Lb. salivarius* and 15 strains of *Lc. lactis* (Supplementary Table 2) were further evaluated by secondary screening. *Lb. salivarius* strains exhibited stronger activity in reducing the expression of joint-related genes than *Lc. Lactis* strains. Moreover, LS2687 was the only strain that significantly reduced the expression of all tested genes compared with the negative control group (Figure 1).

### 3.2 Clinical study

**FIGURE 2:**
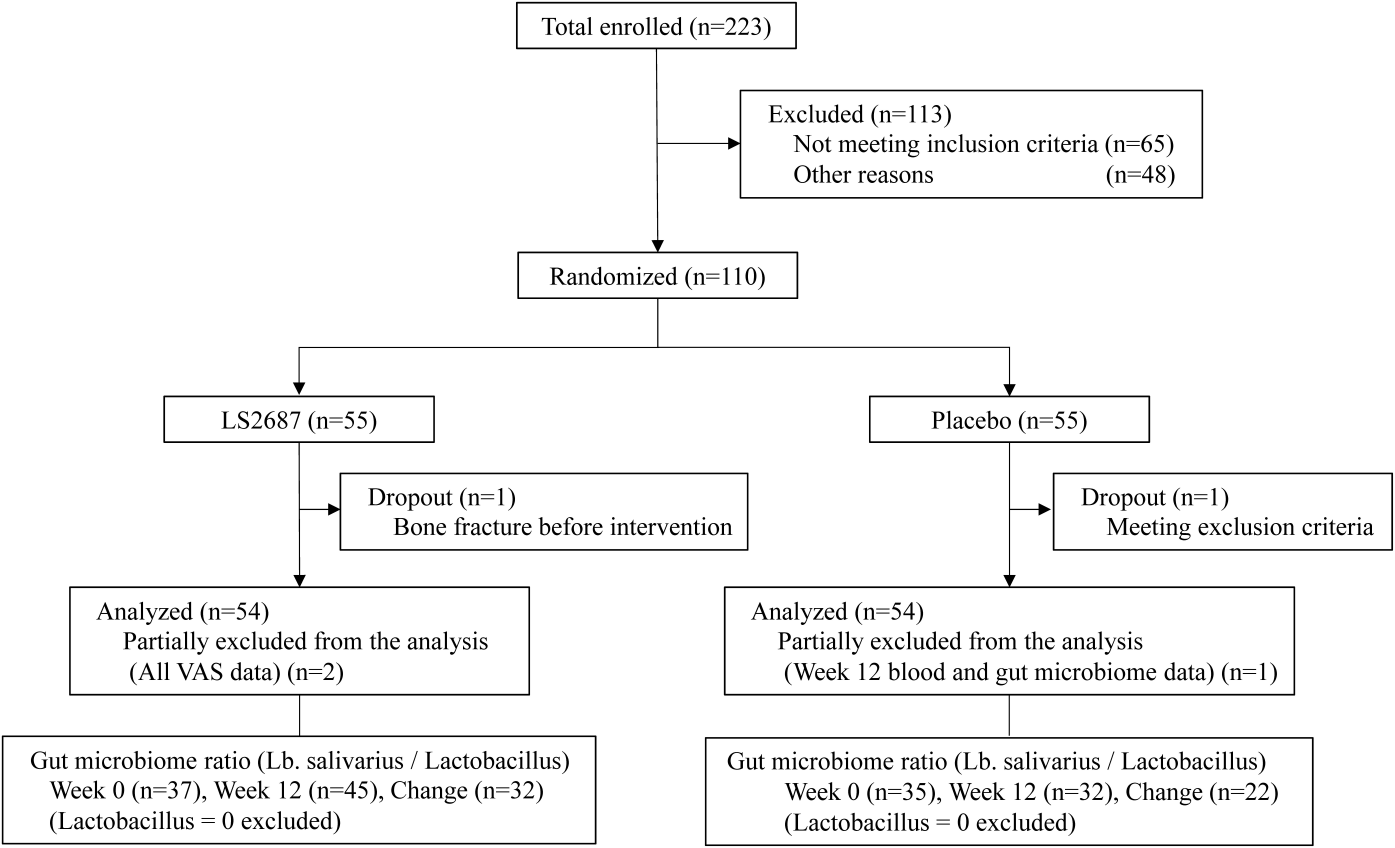
Participant flow diagram. All visual analog scale (VAS) data were excluded because the participants did not report any pain at baseline, precluding assessment of the effect of the intervention. Week 12 blood and gut microbiome data were excluded because of concern regarding potential non-compliance with fasting based on the week 12 blood test results.

Herein, 110 participants were randomized to the LS2687 (55; 50%) and placebo (55; 50%) groups. The analysis population included all randomized participants according to the participant flow diagram (Figure 2). The two participants were dropped out in the study, one participant was once allocated in the LS2687 group but dropped out due to bone fracture before intervention. Another participant was dropped out in the placebo arm due to a past medical history. Adherence rates were 99.6% in the LS2687 group and 99.5% in the placebo group, indicating high adherence to the intervention. The median ages were 53.0 and 51.0 (IQR 46.5–58.0 and 47.0–56.0) years, 36 and 35 (66.7% and 64.8%) were females, and the mean BMIs were 22.7 and 22.6 (IQR 20.8–26.1 and 20.6–24.9) kg/m^2^ in the LS2687 and placebo groups, respectively. The median baseline VAS scores were 3.8 mm (IQR 0.8–8.8), 3.8 mm (IQR 0.08–8.1) and 4.0 mm (IQR 1.1–8.6) for pain, stiffness and discomfort, respectively, in the LS2687 group, and 4.4 mm (IQR 0.5–9.2), 3.2 mm (IQR 0.3–10.5), 3.4 mm (IQR 0.7–12.7), respectively, in the placebo group. The baseline characteristics were similar between the two groups (Table 1).

**TABLE 1:** Study population characteristics assessed at baseline.

Characteristic	LS2687	Placebo	*P*-value
N	54	54	
Sex (female, %)	66.7	64.8	0.839
Age (years)	53.0 (46.5–58.0)	51.0 (47.0–56.0)	0.728
BMI (kg/m^2^)	22.7 (20.8–26.1)	22.6 (20.6–24.9)	0.626
Knee pain, rest (0–100 mm)	3.8 (0.8–8.8)	4.4 (0.5–9.2)	0.838
Knee stiffness, rest (0–100 mm)	3.8 (0.08–8.1)	3.2 (0.3–10.5)	0.957
Knee discomfort, rest (0–100 mm)	4.0 (1.1–8.6)	3.4 (0.7–12.7)	0.967
KL grade, right knee (0/1, n)	3/51	3/51	1.000
KL grade, left knee (0/1, n)	4/50	3/51	1.000
Fasting glucose (mg/mL)	89.0 (85.0–95.0)	90.5 (83.0–97.0)	0.656
HbA1c (%)	5.3 (5.1–5.5)	5.4 (5.1–5.5)	0.843

Values are shown as median (interquartile range) or number (percentage). Data were analyzed by Mann–Whitney *U* test and Pearson’s Chi-squared test, and no significant difference were observed between two groups. BMI, body mass index; VAS, visual analogue scale. A higher score denotes a higher pain intensity. LS2687, *Ligilactobacillus salivarius* SBT2687. KL grade, Kellgren-Lawrence grade. A higher grade indicates worse X-ray findings of the knee. HbA1c, hemoglobin A1c.

### 3.3 Primary outcome

The individual VAS score (in mm) for pain, stiffness and discomfort at wakeup were analyzed as co-primary outcomes, corresponding to pain, discomfort and discomfort at rest, respectively. The mean changes in the VAS score for pain from baseline to the end of the intervention were −2.9 ± 6.1 in the LS2687 group and −1.7 ± 4.8 in the placebo group. Similarly, the mean changes in the VAS score for stiffness were −3.6 ± 7.1 in the LS2687 group and −1.3 ± 5.1 in the placebo group, while the mean changes in the VAS score for discomfort were −3.8 ± 7.5 in the LS2687 group and −1.7 ± 5.3 in the placebo group (Table 2).

**TABLE 2:** Primary and key secondary outcomes at the 12 weeks of intervention periods.

Outcome	*N* ^1^	LS2687	Placebo	*P*-value
Primary outcome				
Δ^2^ VAS pain (0–100)	52/54	−2.9 ± 6.1	−1.7 ± 4.8	0.048*
ΔVAS stiffness (0–100)	52/54	−3.6 ± 7.1	−1.3 ± 5.1	0.014*
ΔVAS discomfort (0–100)	52/54	−3.8 ± 7.5	−1.7 ± 5.3	0.016*
Key secondary outcome				
Subjective Evaluation				
ΔJKOM VAS (0–100)	54/54	−7.6 ± 13.7	−7.7 ± 11.8	0.975
ΔJKOM total (0–100)	54/54	−4.9 ± 5.9	−4.4 ± 4.6	0.494
ΔJOA total (0–100)	54/54	5.7 ± 13.8	4.9 ± 9.8	0.985
Gut microbiome				
*Lactobacillus*, Δ%	54/53	0.41 ± 1.54	0.06 ± 0. 40	0.026*
At 0 week	54/54	0.20 ± 0.68	0. 26 ± 1.10	0.830
At 12 week	54/53	0.61 ± 1.47	0.32 ± 1.43	<0.001***
*Lb salivarius / Lb*^3^, Δ%	32/22	29.16 ± 42.84	−3.75 ± 20.75	0.005**
At 0 week	37/35	3.63 ± 11.30	7.77 ± 23.84	0.940
At 12 week	45/32	35.49 ± 42.47	5.92 ± 20.98	<0.001***
Alpha diversity^4^, Δ	54/53	−0.03 ± 0.32	−0.14 ± 0.51	0.100
At 0 week	54/54	5.27 ± 0.51	5.35 ± 0.60	0.460
At 12 week	54/53	5.24 ± 0.57	5.21 ± 0.58	0.650

Values are shown as means ± SD. Data were analyzed by Mann–Whitney *U* test (*P < 0.05, **P < 0.01, ***P < 0.001). LS2687, *Ligilactobacillus salivarius* SBT2687.

^1^Numbers are expressed as LS2687 / Placebo.

^2^Δ are expressed the change from baseline (week 0) to week 12.

^3^Non-detectors for *Lb.* genus: *N* = 17 and 19 in the LS2687 group and *N* = 9 and 21 in the placebo group at 0 and 12 weeks, respectively.

^4^Shannon–Wiener index

In a group-to-group comparison, the VAS changes appeared to be greater in the LS2687 group throughout the intervention, with comparable and time-dependent decreases in the measurements (*P* < 0.001 vs. baseline). The corresponding between-group difference, 95% confidence interval, and effect size for the primary outcome are shown (Supplementary Table 5). Compared with placebo group, all VAS scores decreased more in the LS2687 group at weeks 6, 7, and 12, and greater decreases were observed at weeks 1 and 8 for stiffness and at weeks 2, 8, and 11 for discomfort (Supplementary Figure 2). Based on these findings, a confirmatory ANCOVA was conducted after adjusting with baseline VAS scores, and resulting that the *P*-values for intervention were 0.24, 0.14, 0.17 and 0.16 in pain, stiffness, discomfort and total, respectively (Supplementary Table 6).

### 3.4 Key secondary outcomes

#### 3.4.1 Subjective assessment

The mean changes in the JKOM and JOA scores improved from baseline in both the LS2687 and placebo groups; however, there was no significant difference between the groups (Table 2). The corresponding between-group differences, 95% confidence intervals, and effect sizes for the key secondary outcomes are presented (Supplementary Table 5).

#### 3.4.2 Gut microbiome analysis

In the LS2687 group, the relative abundance of *Lb. salivarius* within the *Lactobacillus* genus increased from 3.63% to 35.49%, whereas in the placebo group, it was 7.77% to 5.92% (Table 2; Supplementary Figure 3). Among the participants in the LS2687 group, the relative abundance of *Lb. salivarius* increased from 5.53% to 29.44% in responders (VAS scores for pain improved within 12 weeks) and from 0.13% to 39.02% in non-responders (VAS scores for pain did not improve within 12 weeks) (Supplementary Figure 3). The alpha diversity index, based on the Shannon–Wiener index, showed no difference between the LS2687 and placebo groups (Table 2; Supplementary Figure 4).

#### 3.4.3 Safety outcome and adverse events

In this study, 33 adverse events (18 in the LS2687 group and 15 in the placebo group) were reported by 23 participants (12 in the LS2687 group and 11 in the placebo group), none of which were clinically related to the intervention. In addition, blood biochemistry parameters showed no clinically meaningful changes during the intervention (Supplementary Table 7). Although a statistically significant difference was observed in potassium at week 12, this change was not considered clinically meaningful and did not raise any safety concerns. Therefore, there were no safety concerns associated with the consumption of LS2687 for 12 weeks.

#### 3.4.4 Sensitivity analyses and subgroup analyses

The total VAS score for pain, stiffness and discomfort was analyzed as a sensitivity analysis. The mean changes from baseline to the end of the intervention were −10.3 ± 20.0 in the LS2687 group and −4.9 ± 14.3 in the placebo group (*P* = 0.028).

Subgroup analyses were performed as sensitivity analyses for sex, age, BMI, and KL grade (Table 3). Significant within-group effects were observed in the lower BMI range. When participants were stratified by a BMI threshold of 25.0 kg/m^2^, the mean changes in the VAS score for pain in participants with BMI < 25.0 kg/m^2^ were −3.5 ± 6.5 in the LS2687 group and −1.6 ± 4.9 in the placebo group, whereas the mean changes in participants with BMI ≥ 25.0 kg/m^2^ were −3.6 ± 7.0 in the LS2687 group and −3.6 ± 7.0 in the placebo group. Moreover, when participants were stratified by a BMI threshold of 23.0 kg/m^2^, the mean changes in the VAS score for pain in participants with BMI < 23.0 kg/m^2^ were −3.6 ± 7.0 in the LS2687 group and −0.5 ± 4.0 in the placebo group, whereas the mean changes in participants with BMI ≥ 23.0 kg/m^2^ were −2.1 ± 4.9 in the LS2687 group and −3.0 ± 5.4 in the placebo group. A significant trend in the interaction *P*-value was observed only with stratification by a BMI threshold of 23.0 kg/m^2^ (*P* = 0.059). For the effect of baseline in the VAS measurements on the changes of VAS score during the intervention, baseline VAS scores for pain, stiffness and discomfort were significantly associated with changes in each outcome in the univariate regression models (β = −0.549, −0.528 and −0.501, respectively; all *P* < 0.001).

**TABLE 3:** Sensitivity analysis of primary outcomes at the 12 weeks of intervention periods.

Variable	N^1^	VAS knee pain mean change		*P*-value	
		LS2687	Placebo	Within group	Interaction
Overall	52/54	−2.9 ± 6.1	−1.7 ± 4.8	0.048*	
Sex					0.570
Male	18/19	−1.8 ± 2.7	−1.4 ± 4.2	0.740	
Female	34/35	−3.5 ± 7.3	−1.8 ± 5.2	0.270	
Age					0.900
<median	22/29	−2.2 ± 4.0	−1.3 ± 3.6	0.180	
≥median	30/25	−3.4 ± 7.9	−1.6 ± 5.9	0.170	
BMI 25.0					0.350
<25.0 (kg/m^2^)	34/41	−3.5 ± 6.5	−1.6 ± 4.9	0.017*	
≥25.0 (kg/m^2^)	18/13	−3.6 ± 7.0	−3.6 ± 7.0	0.798	
BMI 23.0					0.059
<23.0 (kg/m^2^)	28/28	−3.6 ± 7.0	−0.5 ± 4.0	0.003**	
≥23.0 (kg/m^2^)	24/26	−2.1 ± 4.9	−3.0 ± 5.4	0.946	
KL grade (primary symptomatic knee)					0.760
0	3/3	−3.1 ± 4.5	−0.6 ± 1.0	0.512	
1	49/51	−2.9 ± 6.2	−1.7 ± 5.0	0.063	

Changes in visual analog scale (VAS) scores for pain from baseline to 12 weeks are shown for each subgroup. “*P*-value within group” indicates differences between the LS2687 group and the placebo group within each subgroup, as assessed by the Mann-Whitney *U* test (*P < 0.05, **P < 0.01). “*P*-value for interaction” is based on the *p*-value of the group × variable interaction term from a general linear model; no significant interaction was observed between the two groups. Values are shown as means ± SD. LS2687, *Ligilactobacillus salivarius* SBT2687.

^1^Numbers are expressed as LS2687 / Placebo.

## 4 Discussion

Based on the *in vitro* screening, LS2687 was selected as the probiotic strain for a pre-OA clinical trial. The *in-vitro* screening protocol was performed according to the literature with minor modifications (29). Serum and synovial fluid IL-1β have been reported to increase in patients with OA (30). SW982 is often stimulated with IL-1β in cellular experimental models for OA research (29, 31, 32) Thus, we examined the gene expression levels of *TNF-α*, *MMP-13*, *COX-2*, and *NGF* as OA-related biomarkers. TNF-α is a key proinflammatory cytokine in the pathogenesis of OA and is present in high levels in patients with pre-OA (14, 15). Matrix metalloproteinase (MMP)-13 is a matrix-degrading enzyme and is upregulated from the early stages of OA (33). Cycloxygenase (COX)-2 is an inflammatory pain mediator that contributes to synovial inflammation and promotes a self-perpetuating cycle in the progression of OA (14). Nerve growth factor (NGF) is another pain mediator in OA and is increased in response to inflammatory cytokines (34). Collectively, LS2687 may have modulatory activity in OA-related pathways, based on gene expression in an IL-1β stimulated synovial cell model.

We conducted a clinical study of LS2687 based on the safety profile in the Qualified Presumption of Safety status of *Ligilactobacillus salivarius* (35) and an *in vitro* preclinical study of LS2687 indicated this probiotic strain has no mutagenic activity by the *Ames* test, a widely used, rapid biological assay that determines whether a substance is a mutagen (not published). In this clinical study, 108 participants with knee pre-OA completed a 12-week intervention of daily supplementation with LS2687 or placebo capsules with no apparent adverse effects related to the intervention. There was no significant difference in the frequency of adverse events between the LS2687 and placebo groups, suggesting that supplementation with LS2687 was safe and well-tolerated. The changes in knee pain intensity were similar in both groups from baseline to the end of the intervention, showing a decrease in pain relief throughout the study period, while the pain-relieving effect in the LS2687 group appeared to be greater than that in the placebo group, with respect to the statistical significance observed at some weeks in the group-to-group comparisons. Furthermore, other burdens on the knee joints were relieved – as indicated in a series of VAS measurements – including stiffness and discomfort, consistent with the trend observed for knee pain. In this context, the between-group improvements did not survive baseline adjustment (ANCOVA *P* = 0.14–0.24). Since the participants had border-line symptoms of pre-OA and EsSKOA and did not report apparent symptoms and mechanical changes of knees, the baseline VAS scores were generally low. Accordingly, the observed within-group VAS changes were likely influenced by floor effects, given that participants with near-zero baseline scores have limited room for further decline. In addition, both groups showed within-group improvements, including a modest improvement in the placebo group, suggesting a possible placebo effect. These changes may also be partly due to regression to the mean, as the baseline VAS scores were relatively low. The observed effect sizes were statistically significant but still less than the minimal clinically important difference (MCID) for OA patients (36). In this study, the between-group differences were statistically significant but smaller than the MCID for OA populations. Considering the preventive and exploratory nature of this study, these effect sizes may be relatively small in established OA patients but could still be meaningful in individuals with mild or subclinical symptoms. Aligning with other confounders, BMI was exploratively analyzed in the progression and intervention effects of VAS scores in this study. Although the BMI < 23.0 kg/m^2^ subgroup showed a significant within-group improvement (*P* = 0.003), the BMI × intervention interaction was not significant (*P* = 0.059), so this result should be treated as exploratory and confirmed in a future trial in OA patients. Taken together, these findings should be interpreted as hypothesis-generating, and clinical relevance will be warranted in future studies in OA patients.

Microbiome analysis revealed that the relative abundance of the *Lactobacillus* genus and *Lb. salivarius* within this genus increased in the LS2687 group, suggesting that supplemented probiotics were partly detected in the gut microbiome. However, this increase was not observed in the placebo group. These results implied that dietary intake selectively might modulate the microbiome, potentially enhancing the presence of beneficial probiotic strains. These structural alterations in the gut microbiota may have contributed to the physiological effects of LS2687 supplementation. Previous studies have also reported that the intake of capsules containing *Lactobacillus rhamnosus* GG increased the relative abundance of the *Lactobacillus* genus, supported better sleep, and improved symptoms of depression and anxiety (37). Accordingly, the gut microbiome appeared to be influenced by probiotic supplementation throughout the intervention. Nevertheless, the positive results of *Lb. salivalius* were not consistent between groups from baseline to endpoint and the positive rate would not warrant enough statistical power, resulting in potential bias in the results. The results came from the participants with detectable *Lb.* genus and not most of the study cohort, limiting the implications from the gut microbiome analysis.

In the LS2687 group, the relative abundance of *Lb. salivarius* at 0 week was higher in responders to pain relief than in non-responders. Recent research has shown that differences in baseline gut microbiome composition may be related to individual responses to probiotics intake (38). These findings suggest that LS2687 may have beneficial effects on the gut microbiome, and that individuals with specific gut microbiome profiles may show greater improvement in knee symptoms with LS2687 supplementation.

Previous clinical studies have reported the beneficial effects of probiotics in patients with knee pain. For example, the consumption of *Lactobacillus casei Shirota* improved the Western Ontario and McMaster Universities Osteoarthritis (WOMAC) index and VAS scores (18). Similarly, the consumption of *Streptococcus thermophilus* improved the WOMAC index (19). Previous studies also examined the effects of consuming multiple strains and reported a reduction in WOMAC index and VAS scores while walking (39). These observations support our findings that probiotic supplementation affects gut microbiome composition and may be associated with improvements in knee symptoms. Compared with previous research, this study has several strengths. First, fecal sample analysis indicated that changes in the gut microbiota may be associated with a reduction in knee pain following lactic acid bacteria intake. Symptom severity was evaluated under various conditions by measuring different time points and symptom characteristics. This effect was particularly significant during the rest periods.

This study has some limitations. First, a short intervention may result in limited efficacy in physiological and perceptual changes, considering the chronic nature of knee OA, which requires a longer duration to achieve measurable improvements than the study period. Second, we used 100-mm VAS scores to evaluate knee symptoms instead of the WOMAC pain subscale. We selected the VAS because it uses a 100 mm scale for each item, which is considered more sensitive for observing improvements than the five-point Likert scale used in the WOMAC (40). However, this might lead to issues in comparison of assay sensitivity across studies. Third, although significant improvements were observed in the primary outcome measure (VAS), the effect size was still limited in efficacy and clinical relevance, as well as no significant changes were found in the secondary outcome measures (JKOM and JOA scores). We employed the JKOM and JOA scoring systems to measure the efficacy of intervention in this study, although these systems were developed and accepted for diagnosing OA in Japan (20, 21). Since both the JKOM and JOA are assessment tools originally developed for patients with OA, they may not have been suitable for evaluating knee pain in individuals with pre-OA, as was the case in this study. The participants with milder symptoms and no clinical signs in radiographic criteria could be more applicable for Early Osteoarthritis Questionnaire (41), rather than JKOM and JOA. Magnetic resonance imaging is another plausible option for improved sensitivity (42), although we could not apply this advanced technique due to the issue on limited resources for feasibility in this study. For the concept of gut-joint axis from an extensional discussion of gut microbiome, although this study hypothesized the gut-joint axis, we did not show direct evidence from functional metabolites or inflammatory markers in this study. That brings us additional limitations in this study. Further, additional limitations remained in the *in vitro* screening experiments. We used cell lysates by bead pulverization, which made no apparent outcome in viability-dependence as a function of probiotics. These screening experiments were conducted up to 110 strains × 4 genes at *n* = 3, carrying a multiple-comparisons in Dunnett’s test.

Future research could help address these limitations and shed light on the mechanisms underlying the promising effects of LS2687 on pre-OA in the context of the gut microbiome.

In conclusion, laboratory-selected, probiotic LS2687 supplementation is associated with subjective knee symptoms and the gut microbiome in this study. The dietary supplementation with LS2687 was well tolerated and provided new insights into the probiotic management of adults with knee pre-OA in associations with knee pain, stiffness, and discomfort. The gut microbiome may be involved in the onset and pathogenesis of pre-OA, along with knee symptoms.

## Data Availability

The 16S rRNA sequencing data are available in the SRA online repository as PRJNA1494762 of the accession number and the following URL: https://www.ncbi.nlm.nih.gov/sra/?term=PRJNA1494762. The remaining data is not readily available due to privacy protection of the participants. De-identified data can be made available from the corresponding author upon reasonable request, subject to internal review, potentially including institutional ethical approval and applicable data protection requirements.
