# Supplementary material for: Laboratory-selected, probiotic Ligilactobacillus salivarius SBT2687 supplementation is associated with subjective knee symptoms and the gut microbiome in adults with pre-osteoarthritis: a randomized controlled trial in Japanese
Source: Front Nutr. 2026 Aug 4;13:1863405. doi: 10.3389/fnut.2026.1863405 (PMC13481194; doi:10.3389/fnut.2026.1863405)
Supplement: Supplementary file 1 [file Data_Sheet_1.pdf]

The CONSORT reporting checklist

For checking that reports of randomised trials can be understood and used by everyone

ⓘ Note

If you have not used a reporting guideline before, read about how and why to use them and check whether CONSORT is the most applicable reporting guideline for your work.

Reporting guidelines are most useful when used early in research. When writing a manuscript or application, consider using the Full Guidance where you'll see explanations and examples for each item.

After writing, demonstrate adherence by completing this checklist:

1. Specify where each item is described (see Note 1).
2. Cite this checklist (See Note 2).
3. Include your completed checklist as a supplement when submitting to a journal so that future readers can use it to find information.

	Item Description	Location (or reason for not reporting)
**Title and Abstract**		
1a. Title	Identification as a randomised trial.	Title
1b. Structured Abstract	Structured summary of the trial design, methods, results, and conclusions.	Abstract
**Open Science**		
2. Trial Registration	Name of trial registry, identifying number (with URL) and date of registration.	Registration; Methods – Ethics
Protocol and statistical analysis plan	Where the trial protocol and statistical analysis plan can be accessed.	Methods – Ethics; Methods – Study design and participants; Methods – Statistical analysis (as brief summary from Japanese original documentation)
4. Data sharing	Where and how the individual de-identified participant data (including data dictionary), statistical code and any other materials can be accessed.	Data Availability Statement
5. Funding and Conflicts of Interest		

5a. Funding	Sources of funding and other support (eg, supply of drugs), and role of funders in the design, conduct, analysis, and reporting of the trial.	Funding
5b. Conflicts of interest	Financial and other conflicts of interest of the manuscript authors.	Conflict of Interests
**Introduction**		
6. Background and rationale	Scientific background and rationale.	Introduction
7. Objectives	Specific objectives related to benefits and harms.	Introduction
**Methods**		
8. Patient and public involvement	Details of patient or public involvement in the design, conduct and reporting of the trial.	n/a
9. Trial Design	Description of trial design including type of trial (eg, parallel group, crossover), allocation ratio, and framework (eg, superiority, equivalence, non-inferiority, exploratory).	Methods – Study design and participants
10. Changes to trial protocol	Important changes to the trial after it commenced including any outcomes or analyses that were not pre-specified, with reason.	n/a
11. Trial Setting	Settings (eg, community, hospital) and locations (eg, countries, sites) where the trial was conducted.	Methods – Study design and participants
12. Eligibility Criteria		
12a. Participants	Eligibility criteria for participants.	Methods – Study design and participants
12b. Other	If applicable, eligibility criteria for sites and for individuals delivering the interventions (eg, surgeons, physiotherapists).	n/a, for sites and individuals
13. Intervention and comparator	Intervention and comparator with sufficient details to allow replication. If relevant, where additional materials describing the intervention and comparator (eg, intervention manual) can be accessed.	Methods – Test foods
14. Outcomes	Prespecified primary and secondary outcomes, including the specific measurement variable (eg, systolic blood pressure), analysis metric (eg, change from baseline, final value, time to event), method of aggregation (eg, median, proportion), and time point for each outcome.	Methods – Primary outcomes; Methods – Secondary outcomes
15. Harms	How harms were defined and assessed (eg, systematically, non-systematically).	Methods – Safety analysis
16. Sample Size		

16a. How sample size was determined	How sample size was determined, including all assumptions supporting the sample size calculation.	Methods – Statistical analysis
16b. Interim analyses and stopping criteria	Explanation of any interim analyses and stopping guidelines.	n/a
17. Randomisation		
17a. Sequence Generation	Who generated the random allocation sequence and the method used.	Methods – Randomization and blinding
17b. Type of Randomisation	Type of randomisation and details of any restriction (eg, stratification, blocking, and block size).	Methods – Randomization and blinding
18. Allocation concealment mechanism	Mechanism used to implement the random allocation sequence (eg, central computer/telephone; sequentially numbered, opaque, sealed containers), describing any steps to conceal the sequence until interventions were assigned.	Methods – Randomization and blinding
19. Implementation	Whether the personnel who enrolled and those who assigned participants to the interventions had access to the random allocation sequence.	Methods – Randomization and blinding
20. Blinding		
20a. Who was blinded	Who was blinded after assignment to interventions (eg, participants, care providers, outcome assessors, data analysts).	Methods – Randomization and blinding
20b. How blinding was achieved	If blinded, how blinding was achieved and description of the similarity of interventions.	Methods – Randomization and blinding; Methods – Test foods
21. Statistical methods		
21a. Comparing groups	Statistical methods used to compare groups for primary and secondary outcomes, including harms.	Methods – Statistical analysis
21b. Definition of who is included in each analysis	Definition of who is included in each analysis (e.g., all randomised participants), and in which group.	Methods – Statistical analysis; Figure 2
21c. Missing Data	How missing data were handled in the analysis.	Methods – Statistical analysis
21d. Additional Analyses	Methods for any additional analyses (eg, subgroup and sensitivity analyses), distinguishing pre-specified from post hoc.	Methods – Statistical analysis
22. Participant flow, including flow diagram		
22a. Participant Numbers	For each group, the numbers of participants who were randomly assigned, received intended intervention, and were analysed for the primary outcome.	Fig2

22b. Losses and exclusions	For each group, losses and exclusions after randomisation, together with reasons.	Fig2
23. Recruitment		
23a. Dates	Dates defining the periods of recruitment and follow-up for outcomes of benefits and harms.	n/a (reported in trial registration)
23b. Reasons for stopping recruitment	If relevant, why the trial ended or was stopped.	n/a
24. Intervention and comparator delivery		
24a. As Administered	Intervention and comparator as they were actually administered (eg, where appropriate, who delivered the intervention/comparator, whether participants adhered, whether they were delivered as intended (fidelity)).	Results – Clinical study
24b. Concomitant Care	Concomitant care received during the trial for each group.	n/a
25. Baseline Data	A table showing baseline demographic and clinical characteristics for each group.	Table1
26. Numbers analysed, outcomes, and estimation	For each primary and secondary outcome, by group:<br>• the number of participants included in the analysis.<br>• the number of participants with available data at the outcome time point.<br>• result for each group, and the estimated effect size and its precision (such as 95% confidence interval).<br>• for binary outcomes, presentation of both absolute and relative effect size.	Results – Primary outcome; Results – Key secondary outcomes; Fig2; TableS5
27. Harms	All harms or unintended events in each group.	Results – Safety outcome and adverse events
28. Ancillary Analyses	Any other analyses performed, including subgroup and sensitivity analyses, distinguishing pre-specified from post hoc.	Results – Sensitivity analyses and subgroup analyses; Table3
**Discussion**		
29. Interpretation	Interpretation consistent with results, balancing benefits and harms, and considering other relevant evidence.	Discussion
30. Limitations	Trial limitations, addressing sources of potential bias, imprecision, generalisability, and, if relevant, multiplicity of analyses.	Discussion

1 How to specify where content is

Tell the reader where they can find information. E.g.,

- Results; paragraph 2
- Methods, Participants; paragraphs 1 & 2.
- Table 3
- Supplement B, para. 4

If you have chosen not to describe an item, explain why. You can do this in the checklist, or as a note below it.

You can describe items in the article body, or in tables, figures, or supplementary materials, and should prioritize items you feel are most important to your intended audience. The order of items in your manuscript does not need to match the order of items in this checklist. You can decide how best to structure your work.

2 How to cite

Describe how you used CONSORT at the end of your Methods section, referencing the resources you used e.g.,

> 'We used the CONSORT reporting guideline(1) to draft this manuscript, and the CONSORT reporting checklist(2) when editing, included in supplement A'

If you use a reporting checklist, remember to include it as a supplement when publishing so that readers can easily find information and see how you have interpreted the guidance.

1. Hopewell S, Chan AW, Collins GS, Hróbjartsson A, Moher D, Schulz KF, et al. CONSORT 2025 statement: Updated guideline for reporting randomised trials. The BMJ [Internet]. 2025 Apr;389:e081123. Available from: https://www.ncbi.nlm.nih.gov/pmc/articles/PMC11995449/

2. Hopewell S, Chan AW, Collins GS, Hróbjartsson A, Moher D, Schulz KF, et al. The CONSORT reporting checklist. In: Harwood J, Albury C, Beyer J de, Schlüssel M, Collins G, editors. The EQUATOR network reporting guideline platform [Internet]. The UK EQUATOR Centre; 2025. Available from: https:/resources.equator-network.org/reporting-guidelines/consort/consort-checklist.docx
