# Supplementary material for: Laboratory-selected, probiotic Ligilactobacillus salivarius SBT2687 supplementation is associated with subjective knee symptoms and the gut microbiome in adults with pre-osteoarthritis: a randomized controlled trial in Japanese
Source: Front Nutr. 2026 Aug 4;13:1863405. doi: 10.3389/fnut.2026.1863405 (PMC13481194; doi:10.3389/fnut.2026.1863405)
Supplement: Supplementary file 2 [file Image_1.pdf]

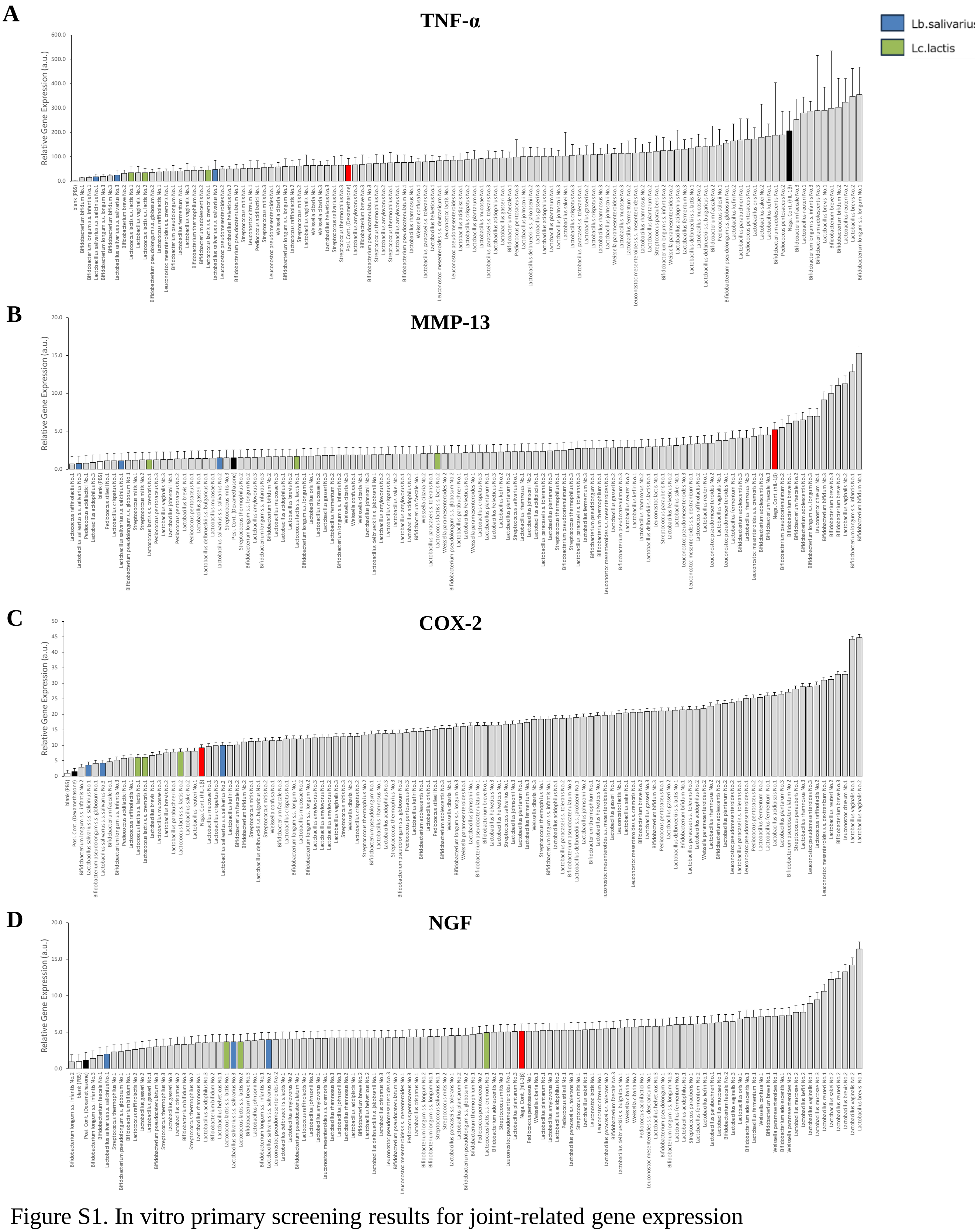

Figure S1. In vitro primary screening results for joint-related gene expression

A total of 110 strains of lactic acid bacteria and bifidobacteria were evaluated for their effects on joint-related gene expression. SW982 cells were treated with IL-1β (1 ng/mL), dexamethasone (100 nM), and lactic acid bacteria samples (1 mg/mL). The expression levels of (A) TNF-α, (B) MMP-13, (C) COX-2, (D) NGF were measured. Data are shown as mean + SD (n=3).

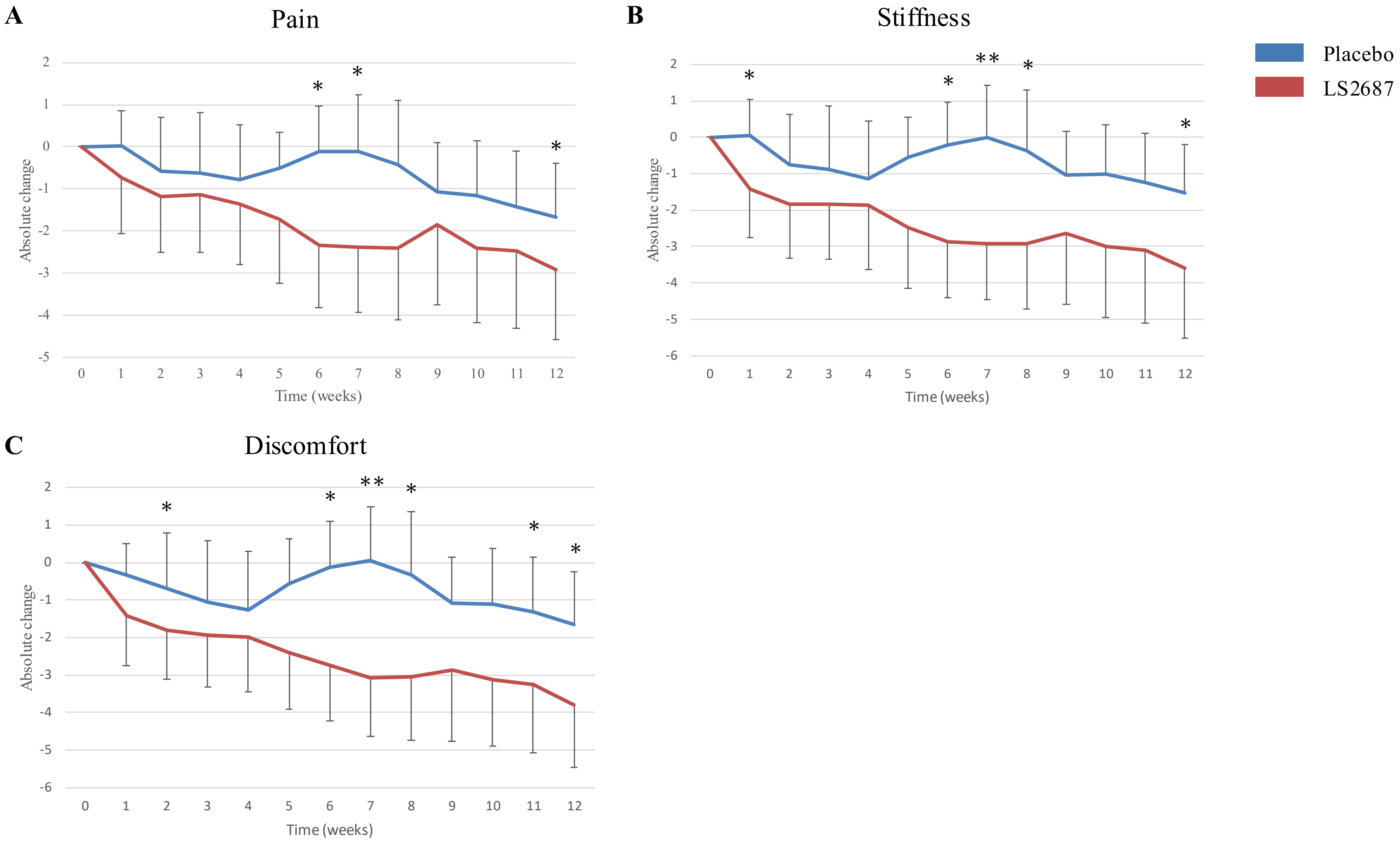

Fig S2. Changes in visual analog scale (VAS) score from baseline.

VAS scores at wake-up for (A) Pain, (B) Stiffness, (C) Discomfort were assessed at each time point. Values are shown as mean changes from baseline. Error bars indicate the 95%CI of the estimates. Statistical analysis was performed by comparing the LS2687 group and the placebo group at each time point using Mann–Whitney *U* test (*$P < 0.05$, **$P < 0.01$). LS2687, *Ligilactobacillus salivarius* SBT2687.

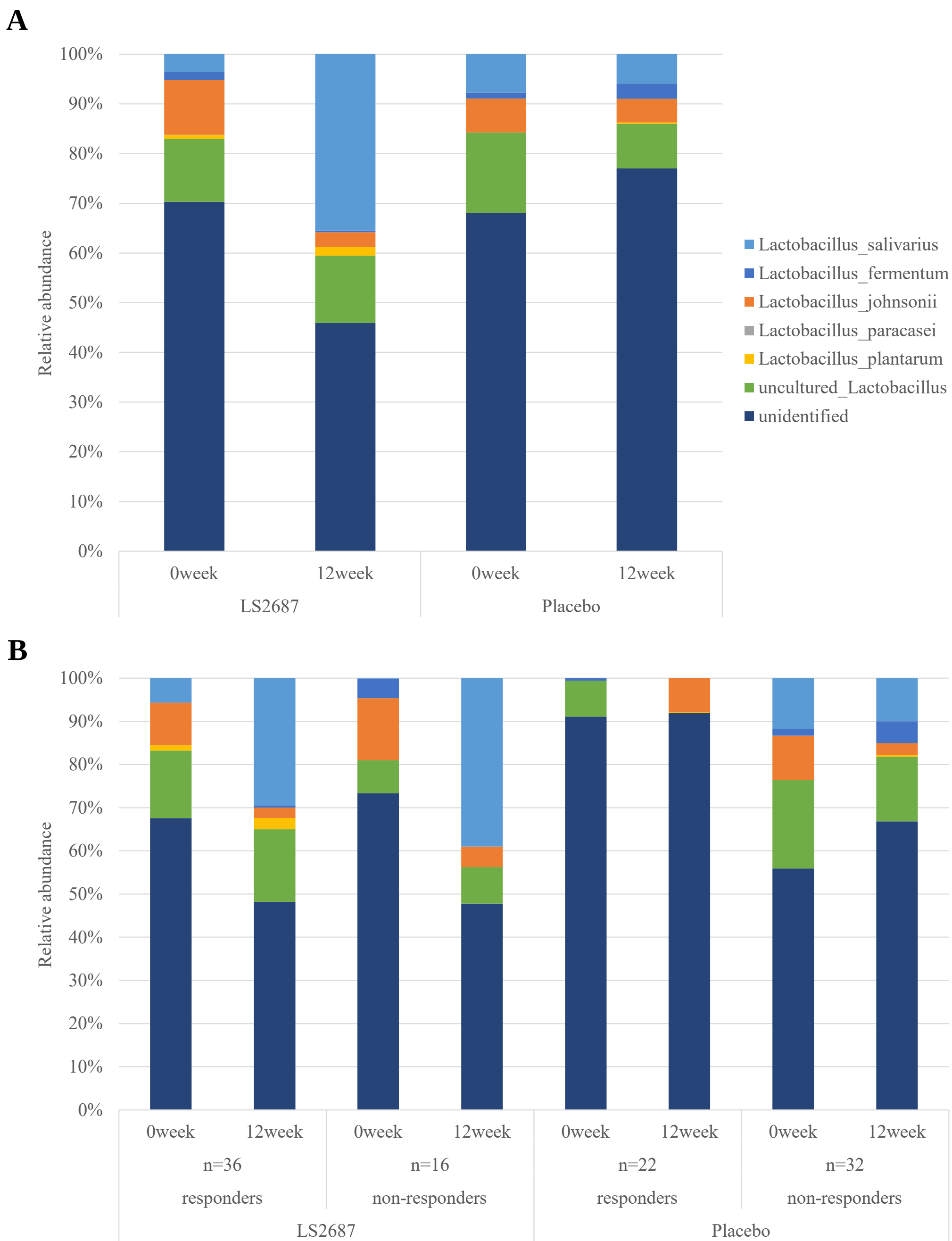

Fig S3. Relative abundance of each species in the *Lactobacillus* genus.
(A) The proportion of each *Lactobacillus* species relative to the total *Lactobacillus* genus was calculated for each participant at baseline and after 12 weeks in the LS2687 group and placebo group. (B) The same analysis as in (A), further divided by responders (VAS scores for pain improved within 12 weeks) and non-responders (VAS scores for pain not improved within 12 weeks). VAS, visual analog scale. LS2687, *Ligilactobacillus salivarius* SBT2687.

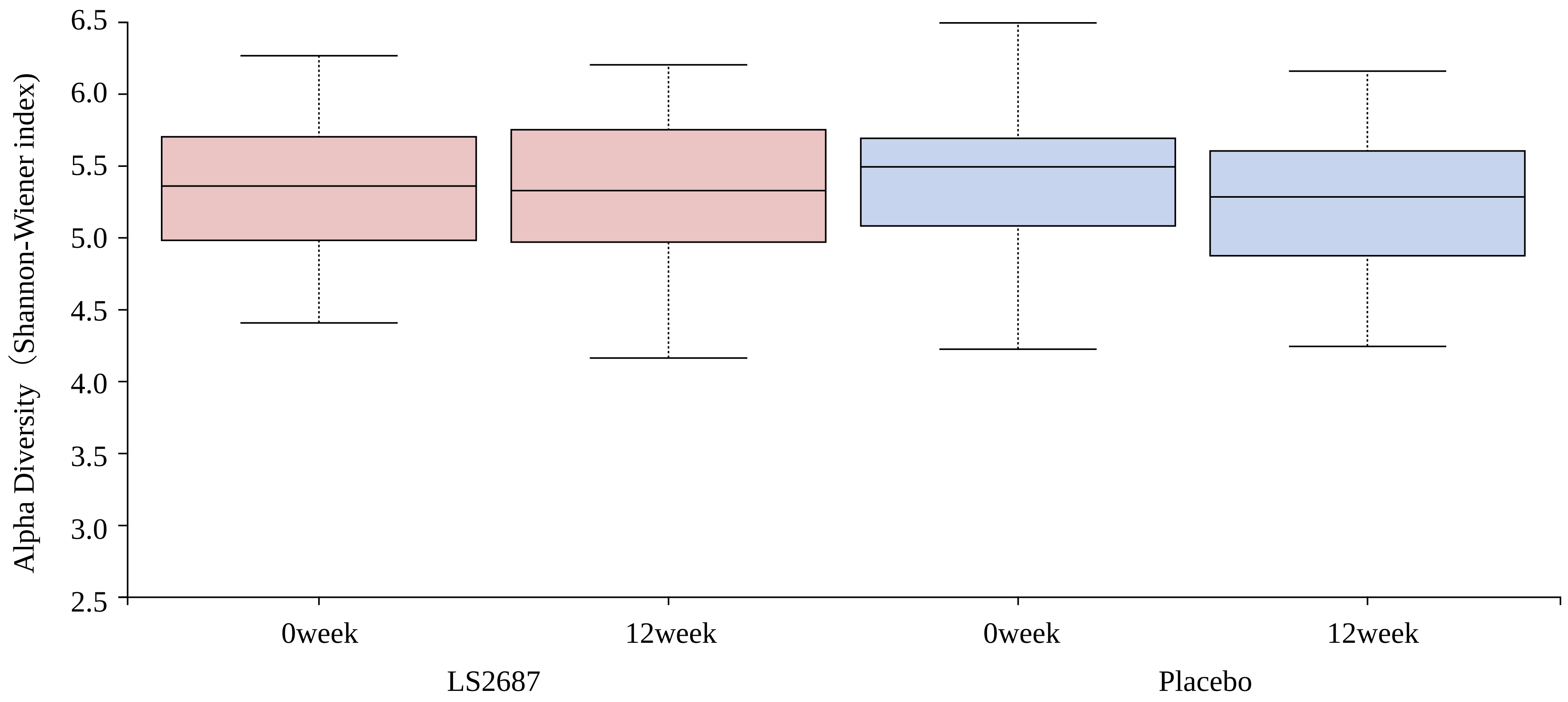

Fig S4. Alpha diversity index between LS2687 group and placebo group.
Alpha diversity was measured by the Shannon-Wiener index. Fecal samples collected at baseline and after 12 weeks from participants in the LS2687 group and placebo group. Values are shown as means ± SD. LS2687, *Ligilactobacillus salivarius* SBT2687.
