# Supplementary material for: Laboratory-selected, probiotic Ligilactobacillus salivarius SBT2687 supplementation is associated with subjective knee symptoms and the gut microbiome in adults with pre-osteoarthritis: a randomized controlled trial in Japanese
Source: Front Nutr. 2026 Aug 4;13:1863405. doi: 10.3389/fnut.2026.1863405 (PMC13481194; doi:10.3389/fnut.2026.1863405)
Supplement: Supplementary file 3 [file Table_1.docx]

***Supplementary Materials***

**1	Supplementary Methods**

**1.1	Materials**

The human synovial cell line SW982 was purchased from the American Type Culture Collection (Manassas, VA, USA). Leibovitz-15 (L-15) medium was purchased from Sigma-Aldrich (St. Louis, MO). Fetal bovine serum (FBS) and a penicillin-streptomycin solution (P/S) were obtained from Thermo Fisher Scientific (Waltham, MA, USA). Zirconia beads were purchased from Promega (Madison, WI, USA), a multi-bead shocker was obtained from Yasui Kikai (Osaka, Japan), and 12-well plates were purchased from IWAKI (Shizuoka, Japan). Interleukin (IL)-1β was obtained from R&D Systems (Minneapolis, MN, USA), and dexamethasone and Sepasol-RNA I Super were purchased from Nacalai Tesque (Kyoto, Japan). The ReverTra Ace qPCR RT Master Mix with gDNA Remover and THUNDERBIRD SYBR qPCR Mix were obtained from Toyobo (Osaka, Japan). Real-time polymerase chain reaction (PCR) was performed using the ViiA™ 7 Real-Time PCR System (Thermo Fisher Scientific, Waltham, MA, USA). All lactic acid bacteria and bifidobacteria were obtained from Megmilk Snow Brand (Tokyo, Japan).

**1.2	Preparation of bacterial samples**

Lactic acid bacteria and bifidobacteria were activated in the optimal culture medium (Tables S1 and S2) and sub-cultured three times. The third subculture was lyophilized and stored at -80 °C until use. On the day of the experiment, the lyophilized bacteria were thawed and pulverized using zirconia beads and a multi-bead shocker. The pulverization program consisted of five cycles at 2,500 rpm for 60 s each, with 60-s intervals between cycles. For the sample preparation, phosphate-buffered saline (PBS) was used to adjust the concentration to 20 mg/mL.

**1.3	Cell culture experiments**

SW982 cells were cultured in L-15 medium containing 10% FBS and 1% P/S. Cells were incubated at 37 °C in a humidified atmosphere containing 5% CO_2_. SW982 cells were seeded at 1.0×10^5^ cells/well in 12-well plates and cultured in L-15 medium containing 10% FBS and 1% P/S for 7 days. On day 4, the culture medium was replaced with fresh L-15 medium containing 10% FBS. After 7 days of culture, the cells were washed with serum-free L-15 medium, and the medium was replaced with fresh serum-free L-15 medium containing human IL-1β at a final concentration of 1 ng/mL to induce inflammation. Then, cell lysates of *Lactobacillus salivarius (Lb. salivarius)* and *Lactococcus lactis (Lc. Lactis)* were added to the IL-1β-stimulated cells at a final concentration of 1 mg/mL. Other groups were prepared as follows: blank (without IL-1β), negative control (with IL-1β), and positive control (with IL-1β and 100 nM dexamethasone). All samples were assayed in triplicate (n=3).

**1.4	Gene expression analysis**

After stimulation for 24 h, total RNA was extracted from SW982 cells using Sepasol RNA I Super. Reverse transcription was performed using ReverTra Ace qPCR RT Master Mix with gDNA Remover, and quantitative real-time PCR was performed using the THUNDERBIRD SYBR qPCR Mix with a ViiA7 Real-time PCR system. The cycling conditions were as follows: 95 °C for 1 min; then 95 °C for 15 s and 60 °C for 30 s; followed by 95 °C for 15 s and 60 °C for 60 s; and finally, 95 °C for 15 s with a temperature ramp rate of 0.05℃/s. Relative gene expression was calculated from standard curves. Glyceraldehyde-3-phosphate dehydrogenase was used as an endogenous control. The sequences of all the primers used in this study are listed in Table S3.

**2	Supplementary Tables**

Table S1. List of bacteria used in the primary screening

| **Species** | **Strain** | **Defined medium** |
| --- | --- | --- |
| *Bifidobacterium adolescentis* | No.1 | GAM+G |
| *Bifidobacterium adolescentis* | No.2 | GAM+G |
| *Bifidobacterium adolescentis* | No.3 | GAM+G |
| *Bifidobacterium bifidum* | No.1 | GAM+G |
| *Bifidobacterium bifidum* | No.2 | GAM+G |
| *Bifidobacterium bifidum* | No.3 | GAM+G |
| *Bifidobacterium breve* | No.1 | GAM+G |
| *Bifidobacterium breve* | No.2 | GAM+G |
| *Bifidobacterium breve* | No.3 | GAM+G |
| *Bifidobacterium faecale* | No.1 | GAM+G |
| *Bifidobacterium faecale* | No.2 | GAM+G |
| *Bifidobacterium faecale* | No.3 | GAM+G |
| *Bifidobacterium longum s.s. longum* | No.1 | GAM+G |
| *Bifidobacterium longum s.s. longum* | No.2 | GAM+G |
| *Bifidobacterium longum s.s. longum* | No.3 | GAM+G |
| *Bifidobacterium longum s.s. infantis* | No.1 | GAM+G |
| *Bifidobacterium longum s.s. infantis* | No.2 | GAM+G |
| *Bifidobacterium longum s.s. infantis* | No.3 | GAM+G |
| *Bifidobacterium pseudocatenulatum* | No.1 | GAM+G |
| *Bifidobacterium pseudocatenulatum* | No.2 | GAM+G |
| *Bifidobacterium pseudocatenulatum* | No.3 | GAM+G |
| *Bifidobacterium pseudolongum* | No.1 | GAM+G |
| *Bifidobacterium pseudolongum s.s. globosum* | No.1 | GAM+G |
| *Bifidobacterium pseudolongum s.s. globosum* | No.2 | GAM+G |
| *Bifidobacterium thermophilum* | No.1 | GAM+G |
| *Lactobacillus acidipiscis* | No.1 | MRS |
| *Lactobacillus acidophilus* | No.1 | MRS |
| *Lactobacillus acidophilus* | No.2 | MRS |
| *Lactobacillus acidophilus* | No.3 | MRS |
| *Lactobacillus amylovorus* | No.1 | MRS |
| *Lactobacillus amylovorus* | No.2 | MRS |
| *Lactobacillus amylovorus* | No.3 | MRS |
| *Lactobacillus brevis* | No.1 | MRS |
| *Lactobacillus brevis* | No.2 | MRS |
| *Lactobacillus crispatus* | No.1 | MRS |
| *Lactobacillus crispatus* | No.2 | MRS |
| *Lactobacillus crispatus* | No.3 | MRS |
| *Lactobacillus delbrueckii s.s. bulgaricus* | No.1 | MRS |
| *Lactobacillus delbrueckii s.s. jakobsenii* | No.1 | MRS |
| *Lactobacillus delbrueckii s.s.lactis* | No.1 | MRS |
| *Lactobacillus fermentum* | No.1 | MRS |
| *Lactobacillus fermentum* | No.2 | MRS |
| *Lactobacillus fermentum* | No.3 | MRS |
| *Lactobacillus gasseri* | No.1 | MRS |
| *Lactobacillus gasseri* | No.2 | MRS |
| *Lactobacillus gasseri* | No.3 | MRS |
| *Lactobacillus helveticus* | No.1 | MRS |
| *Lactobacillus helveticus* | No.2 | MRS |
| *Lactobacillus helveticus* | No.3 | MRS |
| *Lactobacillus johnsonii* | No.1 | MRS |
| *Lactobacillus johnsonii* | No.2 | MRS |
| *Lactobacillus johnsonii* | No.3 | MRS |
| *Lactobacillus kefiri* | No.1 | MRS |
| *Lactobacillus kefiri* | No.2 | MRS |
| *Lactobacillus mucosae* | No.1 | MRS |
| *Lactobacillus mucosae* | No.2 | MRS |
| *Lactobacillus mucosae* | No.3 | MRS |
| *Lactobacillus oris* | No.1 | MRS |
| *Lactobacillus parabuchneri* | No.1 | MRS |
| *Lactobacillus paracasei s.s. tolerans* | No.1 | MRS |
| *Lactobacillus paracasei s.s. tolerans* | No.2 | MRS |
| *Lactobacillus paracasei s.s. tolerans* | No.3 | MRS |
| *Lactobacillus plantarum* | No.1 | MRS |
| *Lactobacillus plantarum* | No.2 | MRS |
| *Lactobacillus plantarum* | No.3 | MRS |
| *Lactobacillus reuteri* | No.1 | MRS |
| *Lactobacillus reuteri* | No.2 | MRS |
| *Lactobacillus rhamnosus* | No.1 | MRS |
| *Lactobacillus rhamnosus* | No.2 | MRS |
| *Lactobacillus rhamnosus* | No.3 | MRS |
| *Lactobacillus sakei* | No.1 | MRS |
| *Lactobacillus sakei* | No.2 | MRS |
| *Lactobacillus salivarius s.s. salicinius* | No.1 | MRS |
| *Lactobacillus salivarius s.s. salivarius* | No.2 | MRS |
| *Lactobacillus salivarius s.s. salivarius* | No.3 | MRS |
| *Lactobacillus vaginalis* | No.1 | MRS |
| *Lactobacillus vaginalis* | No.2 | MRS |
| *Lactobacillus vaginalis* | No.3 | MRS |
| *Lactococcus lactis s.s. lactis* | No.1 | M17 |
| *Lactococcus lactis s.s. lactis* | No.2 | M17 |
| *Lactococcus lactis s.s. cremoris* | No.1 | M17 |
| *Lactococcus raffinolactis* | No.1 | M17 |
| *Lactococcus raffinolactis* | No.2 | M17 |
| *Leuconostoc citreum* | No.1 | MRS |
| *Leuconostoc lactis* | No.1 | MRS |
| *Leuconostoc mesenteroides s.s. cremoris* | No.1 | MRS |
| *Leuconostoc mesenteroides s.s. dextranicum* | No.1 | MRS |
| *Leuconostoc mesenteroides s.s. mesenteroides* | No.1 | MRS |
| *Leuconostoc pseudomesenteroides* | No.1 | MRS |
| *Leuconostoc pseudomesenteroides* | No.2 | MRS |
| *Leuconostoc pseudomesenteroides* | No.3 | MRS |
| *Pediococcus acidilactici* | No.1 | MRS |
| *Pediococcus pentosaceus* | No.1 | MRS |
| *Pediococcus pentosaceus* | No.2 | MRS |
| *Pediococcus pentosaceus* | No.3 | MRS |
| *Pediococcus stilesii* | No.1 | MRS |
| *Streptococcus mitis* | No.1 | MRS(GAM+G) |
| *Streptococcus mitis* | No.2 | MRS(GAM+G) |
| *Streptococcus mitis* | No.3 | MRS(GAM+G) |
| *Streptococcus parauberis* | No.1 | GAM+G |
| *Streptococcus salivarius* | No.1 | MRS |
| *Streptococcus thermophilus* | No.1 | M17+G |
| *Streptococcus thermophilus* | No.2 | M17+G |
| *Streptococcus thermophilus* | No.3 | M17+G |
| *Weissella cibaria* | No.1 | MRS |
| *Weissella cibaria* | No.2 | MRS |
| *Weissella cibaria* | No.3 | MRS |
| *Weissella confusa* | No.1 | MRS |
| *Weissella paramesenteroides* | No.1 | MRS |
| *Weissella paramesenteroides* | No.2 | MRS |

GAM+G: Gifu Anaerobic Medium broth with 1% glucose

MRS: De Man, Rogosa, and Sharpe broth

M17: M17 broth

M17+G: M17 broth with 1% glucose

Table S2. List of *Lactobacillus salivarius* and *Lactococcus lactis* used in the secondary screening

| **Species** | **Strain** | **Defined medium** |
| --- | --- | --- |
| *Lactobacillus salivarius* | No.1 | MRS |
| *Lactobacillus salivarius* | No.2 | MRS |
| *Lactobacillus salivarius* | No.3 | MRS |
| *Lactobacillus salivarius* | No.4 | MRS |
| *Lactobacillus salivarius* | No.5 | MRS |
| *Lactobacillus salivarius* | No.6 | MRS |
| *Lactobacillus salivarius* | No.7 | MRS |
| *Lactobacillus salivarius subsp.salicinius* | No.1 | MRS |
| *Lactobacillus salivarius subsp.salicinius* | No.2 | MRS |
| *Lactobacillus salivarius subsp.salicinius* | No.3 | MRS |
| *Lactobacillus salivarius subsp.salicinius* | No.4 | MRS |
| *Lactobacillus salivarius subsp.salicinius* | No.5 | MRS |
| *Lactobacillus salivarius subsp.salivarius* | No.1 | MRS |
| *Lactobacillus salivarius subsp.salivarius* | No.2 | MRS |
| *Lactobacillus salivarius subsp.salivarius* | No.3 | MRS |
| *Lactobacillus salivarius subsp.salivarius* | No.4 | MRS |
| *Lactobacillus salivarius subsp.salivarius* | No.5 | MRS |
| *Lactobacillus salivarius subsp.salivarius* | No.6 | MRS |
| *Lactobacillus salivarius subsp.salivarius* | No.7 | MRS |
| *Lactobacillus salivarius subsp.salivarius* | No.8 | MRS |
| *Lactobacillus salivarius subsp.salivarius* | No.9 | MRS |
| *Lactobacillus salivarius subsp.salivarius* | No.10 | MRS |
| *Lactobacillus salivarius subsp.salivarius* | SBT 2687 | MRS |
| *Lactobacillus salivarius subsp.salivarius* | No.11 | MRS |
| *Lactobacillus salivarius subsp.salivarius* | No.12 | MRS |
| *Lactobacillus salivarius subsp.salivarius* | No.13 | MRS |
| *Lactococcus lactis* | No.1 | M17 |
| *Lactococcus lactis* | No.2 | M17 |
| *Lactococcus lactis* | No.3 | M17 |
| *Lactococcus lactis* | No.4 | M17 |
| *Lactococcus lactis subsp. cremoris* | No.1 | M17 |
| *Lactococcus lactis subsp. cremoris* | No.2 | M17 |
| *Lactococcus lactis subsp. cremoris* | No.3 | M17 |
| *Lactococcus lactis subsp. cremoris* | No.4 | M17 |
| *Lactococcus lactis subsp. cremoris* | No.5 | M17 |
| *Lactococcus lactis subsp. cremoris* | No.6 | M17 |
| *Lactococcus lactis subsp. cremoris* | No.7 | M17 |
| *Lactococcus lactis subsp. cremoris* | No.8 | M17 |
| *Lactococcus lactis subsp. cremoris* | No.9 | M17 |
| *Lactococcus lactis subsp.lactis* | No.1 | M17 |
| *Lactococcus lactis subsp.lactis* | No.2 | M17 |

MRS: De Man, Rogosa, and Sharpe broth

M17: M17 broth

Table S3. List of qPCR primers

| Gene name | Species |  | Sequence | | | bp |
| --- | --- | --- | --- | --- | --- | --- |
| GAPDH | Human | Forward | 5’ | CTGGGCTACACTGAGCACC | 3’ | 19 |
|  |  | Reverse | 5’ | AAGTGGTCGTTGAGGGCAATG | 3’ | 21 |
| TNF-α | Human | Forward | 5’ | GAGGCCAAGCCCTGGTATG | 3’ | 19 |
|  |  | Reverse | 5’ | CGGGCCGATTGATCTCAGC | 3’ | 19 |
| MMP-13 | Human | Forward | 5’ | TCCTGATGTGGGTGAATACAATG | 3’ | 23 |
|  |  | Reverse | 5’ | GCCATCGTGAAGTCTGGTAAAAT | 3’ | 23 |
| COX-2 | Human | Forward | 5’ | ATGCTGACTATGGCTACAAAAGC | 3’ | 23 |
|  |  | Reverse | 5’ | TCGGGCAATCATCAGGCAC | 3’ | 19 |
| NGF | Human | Forward | 5’ | GGCAGACCCGCAACATTACT | 3’ | 20 |
|  |  | Reverse | 5’ | CACCACCGACCTCGAAGTC | 3’ | 19 |

Table S4. Composition of the test food per 3 capsules (daily intake amount).

|  | LS2687 | Placebo |
| --- | --- | --- |
| LS2687 (cells) | 1.58×10^11^ | 0 |
| Energy (kcal) | 3.63 | 3.10 |
| Protein (g) | 0.19 | 0.00 |
| Fat (g) | 0.03 | 0.02 |
| Carbohydrates (g) | 0.64 | 0.73 |
| Sodium (g) | 0.00 | 0.00 |

LS2687: *Ligilactobacillus salivarius* SBT2687.

Table S5. Between-group differences in primary and key secondary outcomes at the 12 weeks of intervention periods.

|  | Mean difference (95％CI) | Cohen's d |
| --- | --- | --- |
| *Primary outcome* |  |  |
| Δ^1^VAS pain (0-100) | -1.25 (-3.36 to 0.87) | -0.227 |
| ΔVAS stiffness (0-100) | -2.06 (-4.41 to 0.30) | -0.225 |
| ΔVAS discomfort (0-100) | -2.13 (-4.64 to 0.37) | -0.257 |
| *Key secondary outcome* |  |  |
| *Subjective Evaluation* |  |  |
| ΔJKOM VAS (0-100) | 0.13 (-4.74 to 5.00) | 0.010 |
| ΔJKOM total (0-100) | -0.50 (-2.51 to 1.51) | -0.095 |
| ΔJOA total (0-100) | 0.83 (-3.74 to 5.40) | 0.070 |
| *Gut microbiome* |  |  |
| *Lactobacillus,* Δ％ | -0.35 (-0.09 to 0.78) | 0.307 |
| At 0week | -0.06 (-0.41 to 0.29) | -0.068 |
| At 12week | -0.29 (-0.27 to 0.85) | 0.200 |
| *Lb salivarius / Lb*, Δ％ | 0.27 (0.0045 to 0.54) | 0.390 |
| At 0week | -0.02 (-0.04 to 0.03) | -0.015 |
| At 12week | 0.27 (0.0041 to 0.54) | 0.389 |
| Alpha diversity^2^, Δ | 0.11 (-0.05 to 0.27) | 0.255 |
| At 0week | -0.08 (-0.29 to 0.13) | -0.146 |
| At 12week | 0.03 (-0.19 to 0.25) | 0.050 |

^1^ Δ are expressed the change from baseline (week 0) to week 12.

^2^ Shannon-Wiener index

Table S6. Results of ANCOVA for VAS outcomes adjusting for baseline values.

| Outcome | Adjusted mean difference (95% CI) | *P*-value |
| --- | --- | --- |
| VAS pain (0-100) | -1.05 (-2.84 to 0.73) | 0.24 |
| VAS stiffness (0-100) | -1.51 (-3.54 to 0.51) | 0.14 |
| VAS discomfort (0-100) | -1.54 (-3.73 to 0.66) | 0.17 |
| VAS total (0-300) | -4.14 (-9.91 to 1.62) | 0.16 |

Values represent adjusted mean differences between groups (LS2687 minus placebo) with 95% confidence intervals, based on ANCOVA adjusting for baseline VAS values. LS2687, *Ligilactobacillus salivarius* SBT2687.

Table S7. Summary of blood biochemistry parameters.

|  |  | LS2687 | Placebo | *P*-value |
| --- | --- | --- | --- | --- |
| WBC (×10³/μL) | Week0 | 5.34 ± 1.14 | 5.05 ± 1.09 | 0.190 |
|  | Week12 | 5.39 ± 1.23 | 4.93 ± 1.20 | 0.055 |
| RBC (×10⁴/μL) | Week0 | 460 ± 39 | 459 ± 45 | 0.879 |
|  | Week12 | 458 ± 37 | 457 ± 45 | 0.887 |
| Hemoglobin (g/dL) | Week0 | 14.0 ± 1.2 | 14.0 ± 1.3 | 0.796 |
|  | Week12 | 13.7 ± 1.1 | 13.7 ± 1.3 | 0.879 |
| Hematocrit (%) | Week0 | 41.7 ± 3.2 | 41.6 ± 3.6 | 0.853 |
|  | Week12 | 41.9 ± 3.0 | 41.7 ± 3.4 | 0.753 |
| Platelet count (×10⁴/μL) | Week0 | 27.7 ± 5.8 | 26.4 ± 5.7 | 0.220 |
|  | Week12 | 26.7 ± 5.3 | 25.4 ± 4.9 | 0.182 |
| MCV (fL) | Week0 | 91 ± 4 | 91 ± 4 | 0.875 |
|  | Week12 | 92 ± 4 | 92 ± 4 | 0.908 |
| MCH (pg) | Week0 | 30.6 ± 1.3 | 30.5 ± 1.4 | 0.874 |
|  | Week12 | 29.9 ± 1.4 | 29.9 ± 1.5 | 0.976 |
| MCHC (%) | Week0 | 33.7 ± 0.7 | 33.6 ± 0.8 | 0.673 |
|  | Week12 | 32.7 ± 0.7 | 32.7 ± 0.9 | 0.765 |
| Total protein (g/dL) | Week0 | 7.1 ± 0.3 | 7.0 ± 0.3 | 0.824 |
|  | Week12 | 7.0 ± 0.3 | 6.9 ± 0.4 | 0.668 |
| Albumin (g/dL) | Week0 | 4.3 ± 0.2 | 4.4 ± 0.2 | 0.639 |
|  | Week12 | 4.3 ± 0.2 | 4.3 ± 0.2 | 0.470 |
| Albumin/Globulin ratio | Week0 | 1.6 ± 0.2 | 1.6 ± 0.2 | 0.564 |
|  | Week12 | 1.7 ± 0.2 | 1.7 ± 0.2 | 0.819 |
| ALP (U/L) | Week0 | 73 ± 19 | 68 ± 14 | 0.094 |
|  | Week12 | 71 ± 17 | 67 ± 13 | 0.187 |
| AST (U/L) | Week0 | 22 ± 7 | 21 ± 6 | 0.542 |
|  | Week12 | 21 ± 6 | 21 ± 5 | 0.767 |
| ALT (U/L) | Week0 | 23 ± 16 | 21 ± 12 | 0.522 |
|  | Week12 | 20 ± 14 | 21 ± 12 | 0.762 |
| LDH (U/L) | Week0 | 175 ± 29 | 170 ± 25 | 0.305 |
|  | Week12 | 170 ± 26 | 170 ± 28 | 0.989 |
| γ-GTP (U/L) | Week0 | 31 ± 25 | 28 ± 22 | 0.530 |
|  | Week12 | 27 ± 19 | 28 ± 25 | 0.680 |
| Total bilirubin (mg/dL) | Week0 | 0.8 ± 0.2 | 0.8 ± 0.3 | 0.941 |
|  | Week12 | 0.7 ± 0.3 | 0.7 ± 0.3 | 0.501 |
| Total cholesterol (mg/dL) | Week0 | 220 ± 33 | 218 ± 33 | 0.680 |
|  | Week12 | 217 ± 34 | 214 ± 32 | 0.590 |
| LDL cholesterol (mg/dL) | Week0 | 126 ± 29 | 124 ± 28 | 0.741 |
|  | Week12 | 123 ± 30 | 121 ± 28 | 0.761 |
| HDL cholesterol (mg/dL) | Week0 | 70 ± 17 | 70 ± 18 | 0.898 |
|  | Week12 | 70 ± 17 | 69 ± 16 | 0.768 |
| Triglycerides (mg/dL) | Week0 | 100 ± 54 | 87 ± 48 | 0.193 |
|  | Week12 | 92 ± 52 | 93 ± 49 | 0.926 |
| Sodium (mEq/L) | Week0 | 141 ± 1 | 141 ± 2 | 0.547 |
|  | Week12 | 140 ± 1 | 140 ± 2 | 0.788 |
| Potassium (mEq/L) | Week0 | 4.3 ± 0.3 | 4.3 ± 0.3 | 0.223 |
|  | Week12 | 4.2 ± 0.3 | 4.1 ± 0.3 | 0.047* |
| Chloride (mEq/L) | Week0 | 104 ± 2 | 104 ± 2 | 0.814 |
|  | Week12 | 104 ± 2 | 104 ± 2 | 0.446 |
| Calcium (mg/dL) | Week0 | 9.4 ± 0.3 | 9.4 ± 0.3 | 0.679 |
|  | Week12 | 9.2 ± 0.3 | 9.3 ± 0.2 | 0.673 |
| Magnesium (mg/dL) | Week0 | 2.2 ± 0.2 | 2.2 ± 0.2 | 0.911 |
|  | Week12 | 2.3 ± 0.2 | 2.3 ± 0.2 | 0.497 |
| Phosphorus (mg/dL) | Week0 | 3.4 ± 0.4 | 3.4 ± 0.5 | 0.782 |
|  | Week12 | 3.3 ± 0.4 | 3.4 ± 0.5 | 0.308 |
| Blood urea nitrogen (mg/dL) | Week0 | 13.2 ± 3.3 | 12.9 ± 3.5 | 0.645 |
|  | Week12 | 13.6 ± 3.5 | 12.5 ± 3.0 | 0.098 |
| Creatinine (mg/dL) | Week0 | 0.73 ± 0.15 | 0.70 ± 0.14 | 0.279 |
|  | Week12 | 0.80 ± 0.15 | 0.78 ± 0.16 | 0.417 |
| Uric acid (mg/dL) | Week0 | 5.1 ± 1.4 | 5.0 ± 1.2 | 0.534 |
|  | Week12 | 5.2 ± 1.4 | 4.9 ± 1.2 | 0.314 |
| Fasting glucose (mg/mL) | Week0 | 92 ± 7 | 91 ± 9 | 0.655 |
|  | Week12 | 92 ± 8 | 90 ± 9 | 0.183 |
| HbA1c (%) | Week0 | 5.3 ± 0.3 | 5.3 ± 0.3 | 0.843 |
|  | Week12 | 5.3 ± 0.3 | 5.3 ± 0.4 | 0.584 |
| CPK (U/L) | Week0 | 126 ± 81 | 105 ± 48 | 0.104 |
|  | Week12 | 131 ± 90 | 113 ± 56 | 0.205 |

Values are shown as means ± SD. Data were analyzed by unpaired t-test (**P* < 0.05). LS2687, *Ligilactobacillus salivarius* SBT2687. Numbers are expressed as LS2687 / placebo. The number of participants was 54/54 at week 0 and 54/53 at week 12.
